# Supplementary material for: Mature and migratory dendritic cells promote immune infiltration and response to anti-PD-1 checkpoint blockade in metastatic melanoma
Source: Nat Commun. 2025 Sep 1;16:8151. doi: 10.1038/s41467-025-62878-5 (PMC12402436; doi:10.1038/s41467-025-62878-5)
Supplement: Supplementary file 1 — Supplementary Information [file 41467_2025_62878_MOESM1_ESM.pdf]

Supplementary Figure 1.

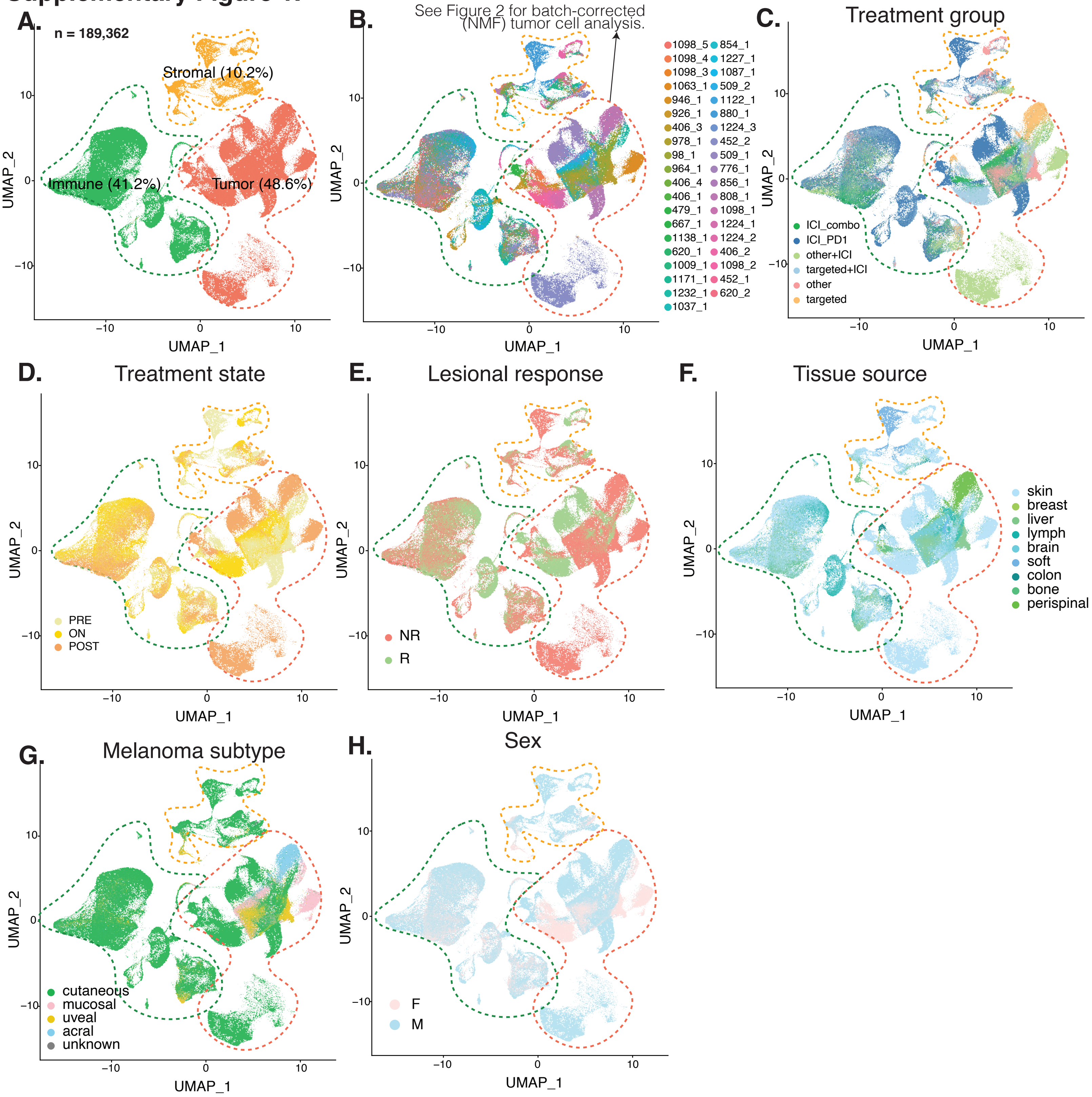

Supplementary Figure 1.Visualization of the scRNA-seq atlas. Uniform manifold approximation and projection (UMAP) embedding of 189,362 cells from the 39 samples, after quality control, with each color representing a compartment (A), a sample (B), a treatment group (C), a treatment state (D), lesional response to treatment (E), a tissue source (F), a melanoma subtype (G), and sex (H). ICI, immune check-point inhibitor; ICI combo, anti-PD1 plus anti-CTLA4; NR, non-responder; R, responder.

Supplementary Figure 2.

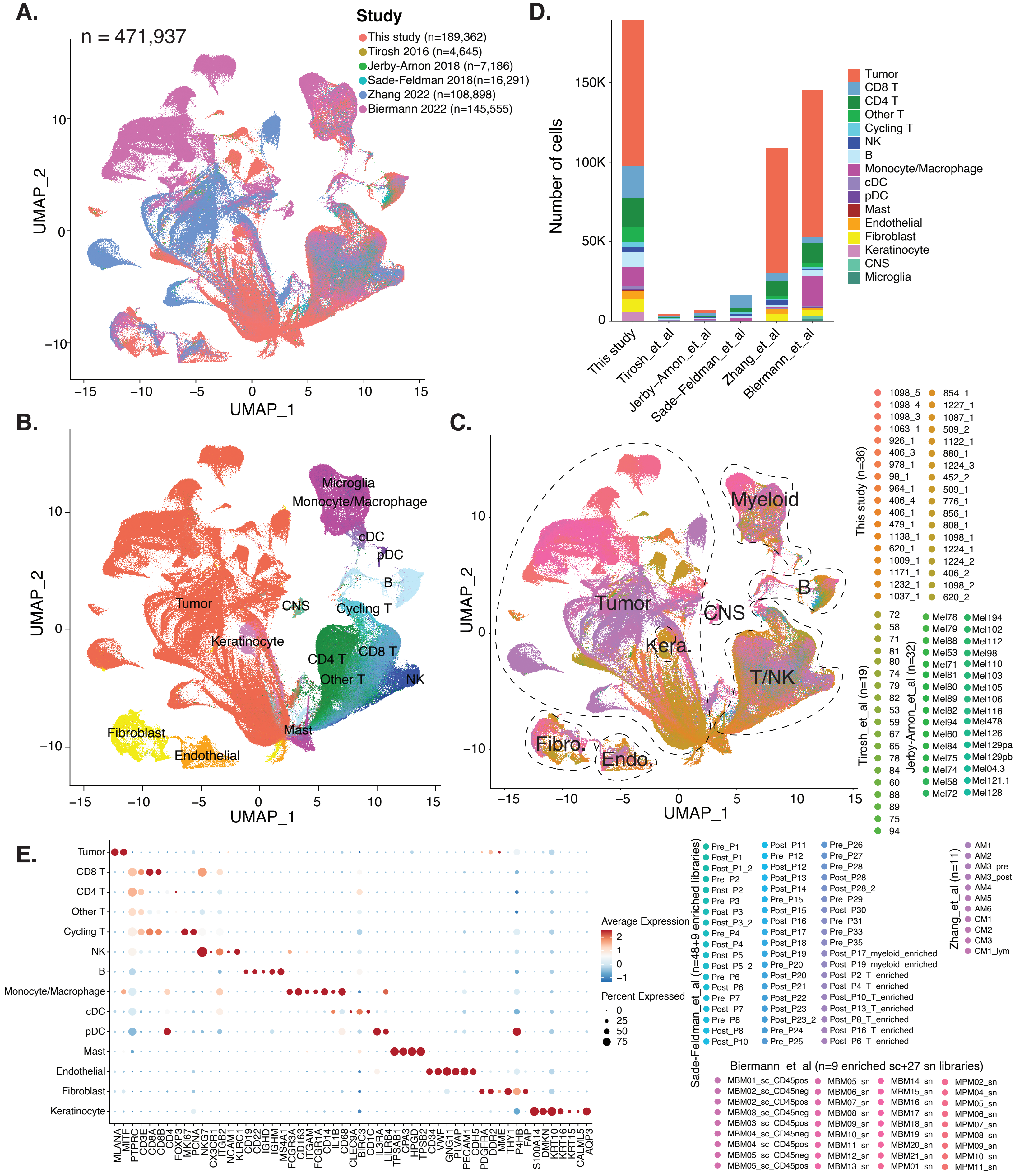

Supplementary Figure 2. A six-study metastatic melanoma single-cell atlas. We integrated our single-cell dataset with five published single-cell datasets (Tirosh et al.[1], Jerby-Arnon et al.[2], Sade-Feldman et al.[3], Zhang et al.[4], and Biermann et al.[5]). Uniform manifold approximation and projection (UMAP) embedding of 471,937 cells from the six studies of 191 samples, with each color representing a study (A), a cell type (B), and a sample (C). D, Distribution of cells by cell types across different studies. E, Dotplot showing average expression and percent expression of marker genes across all the cells from the six studies.

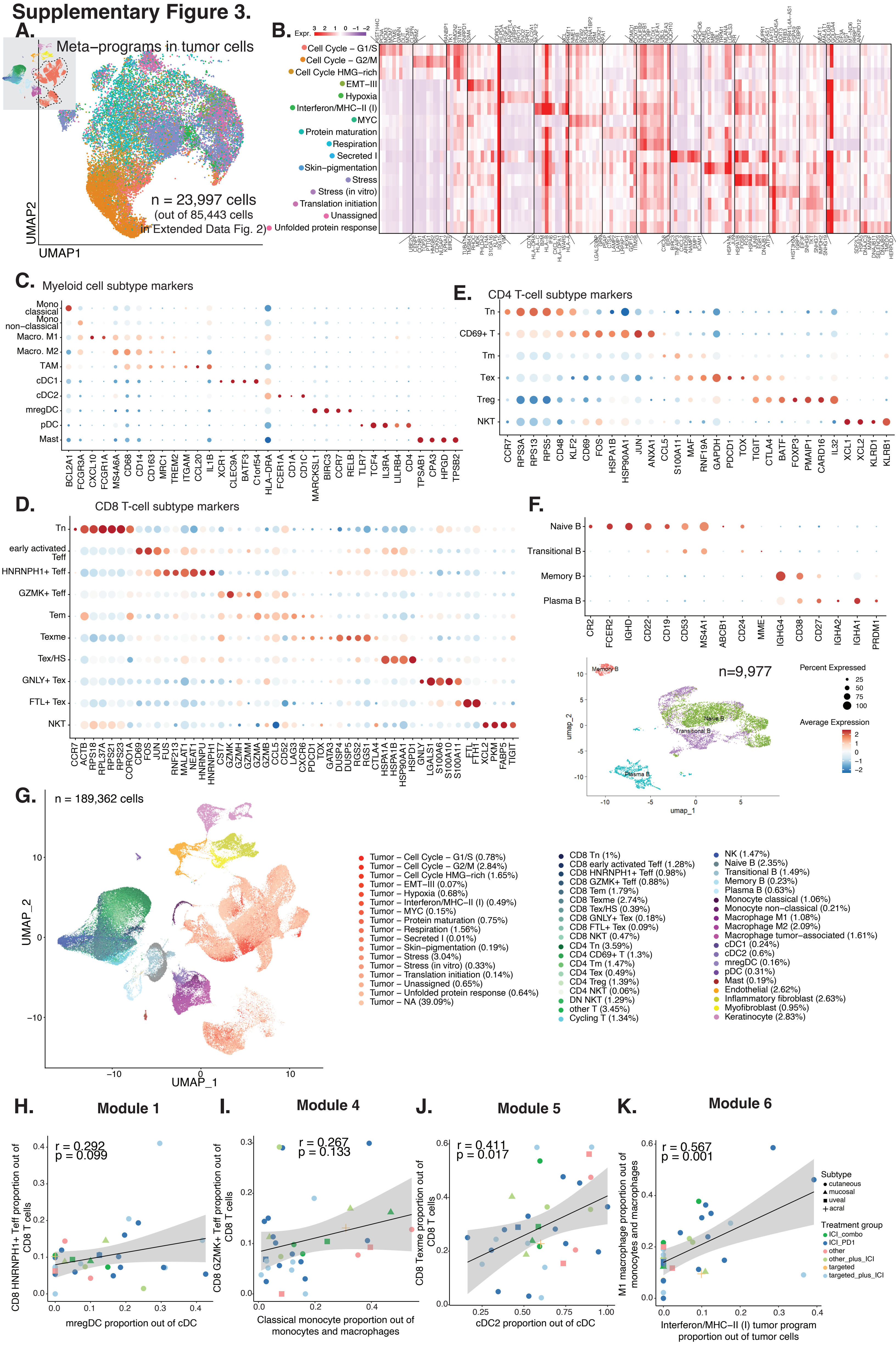

Supplementary Figure 3. Tumor cell programs, cell subtype markers and pairwise correlations between certain cell subtypes. A, UMAP embedding of 23,997 tumor cells from 31 samples annotated with meta-programs according to Gavish et al.[6] B, Heatmap of the top marker genes for each of the identified tumor meta-programs. C-E, Dotplot showing average expression and percent expression of marker genes for myeloid cell subtypes (C), CD8 T-cell subtypes (D), and CD4 T-cell subtypes (E). F, Dotplot showing average expression and percent expression of marker genes for B-cell subtypes (top) and UMAP embedding of subtypes of 9,977 B-cells from 33 samples (bottom). G, UMAP embedding of 189,362 cells from 36 samples colored by cell subtype. H-K, Scatterplots depicting the pairwise correlations between two cell subtypes within modules 1 (H), 4 (I), 5 (J), and 6 (K). Each data point represents a sample, distinguished by shape representing melanoma subtype and color indicating the treatment group. There are 35 samples included in this analysis. Pearson correlation coefficients and p-values are provided within each plot.

Supplementary Figure 4.

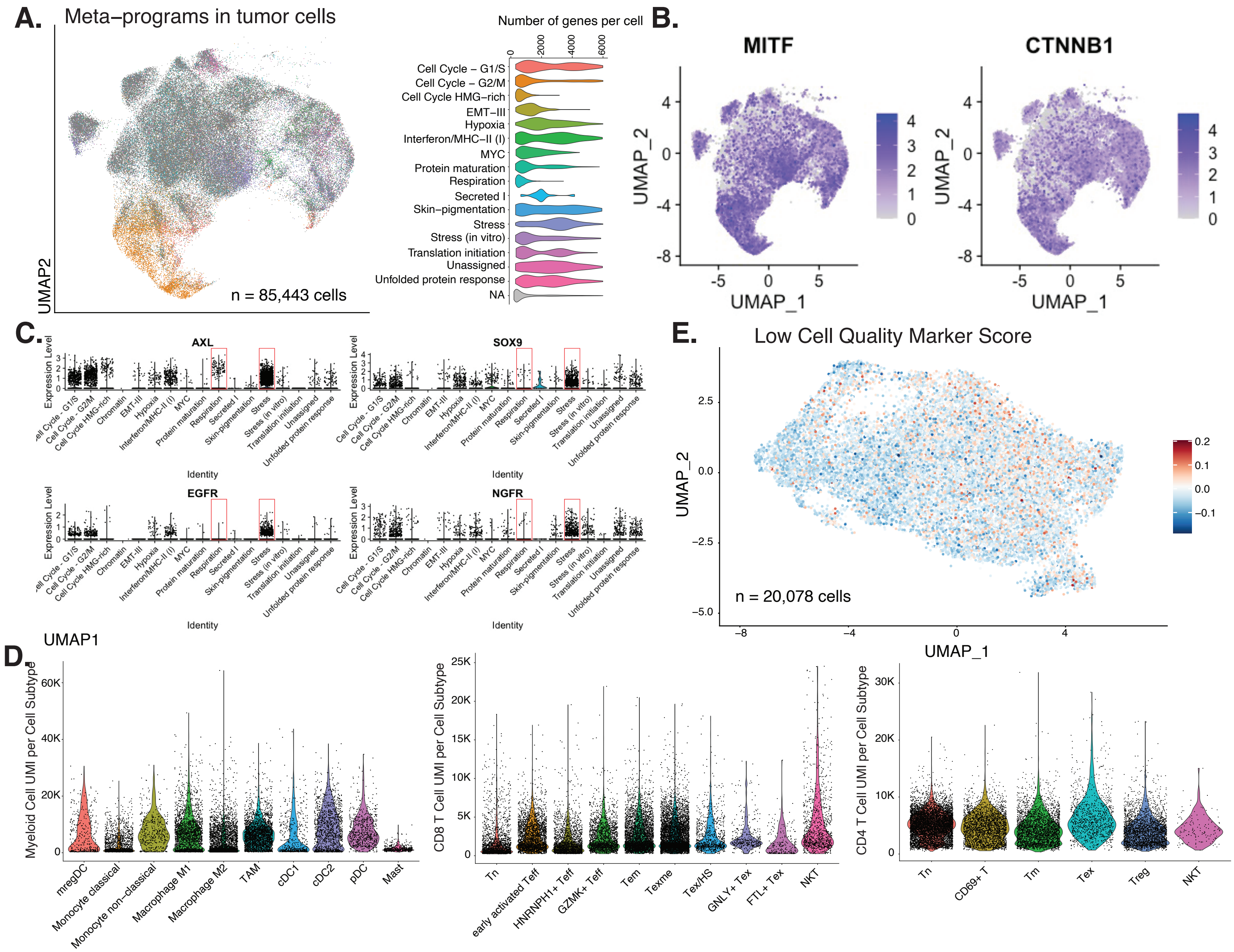

Supplementary Figure 4. Quality control metrics and marker gene expression patterns of tumor cells, -cells and myeloid cells. A, On the left, UMAP embedding of 85,443 tumor cells from 31 samples. Each color represents either a specific tumor meta-program or cells that did not pass the quality control in this analysis. On the right, violin plots displaying the number of genes per cell, categorized by their inclusion in different tumor meta-programs or unannotated cells. B, Tumor cell expression patterns of two melanocytic markers, MITF and CTNNB1, on the UMAP. C, Violin plots showing expression levels of four undifferentiated and dedifferentiated melanoma markers across tumor meta-programs. The respiration program resembles an undifferentiated state while the stress program reflects a dedifferentiated neural crest-like state. D, The number of UMIs per cell distributions for myeloid cells (left), CD8 T-cells (middle) and CD4 T-cells (right). E, Low cell quality marker score for 20,078 CD8 T-cells from 33 samples as represented on the UMAP. The score was calculated based on markers provided in Rich et al.[7]

Supplementary Figure 5.

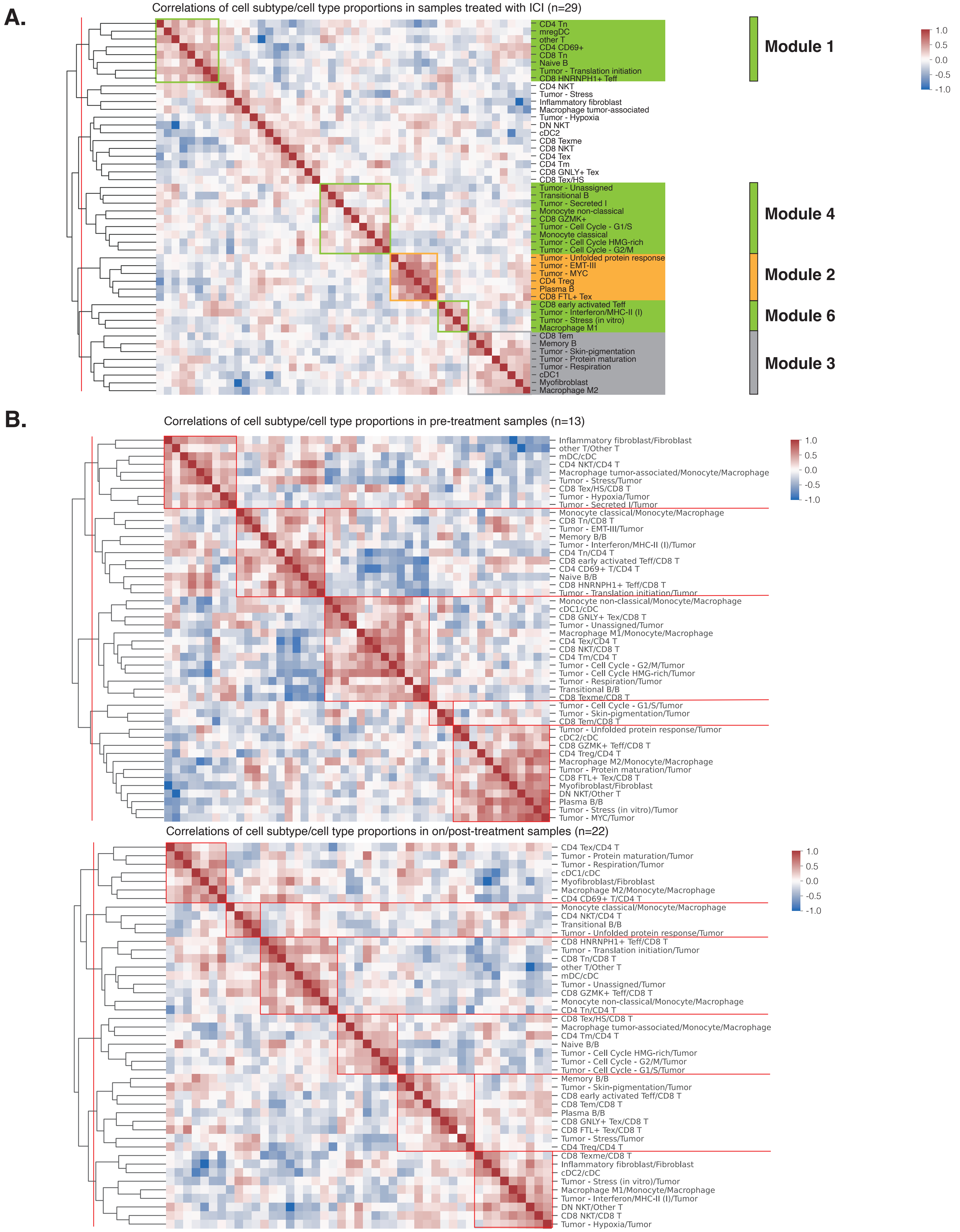

Supplementary Figure 5. Cell subtype proportion correlations for subset of samples. A,B, Heatmap illustrating correlation coefficients among the relative proportions of cell subtypes in relation to their corre-sponding cell types across 29 samples treated with ICI (A), 13 samples collected pre-treatment (B), and 22 samples collected on- or post-treatment (C). We identified clusters/modules by segmenting the hierarchical clustering tree at the red line and assigned annotations based on the modules discovered in Fig. 2D.

Supplementary Figure 6.

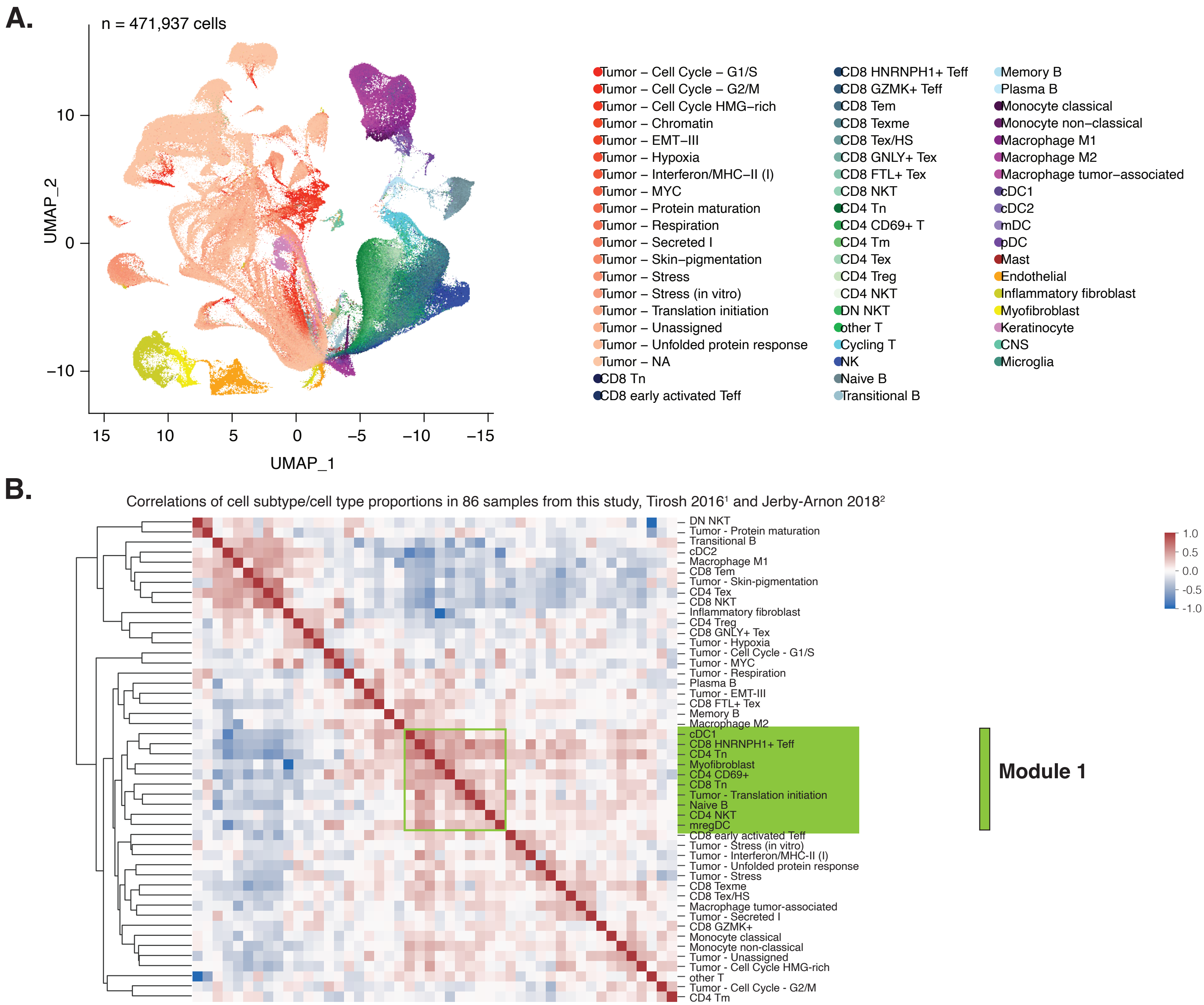

Supplementary Figure 6. UMAP representation and cell subtype proportion correlations for the five-study single-cell atlas. A, UMAP embedding of 471,937 cells from 191 samples in six studies, with each color repre-senting a subtype as annotated in this study. B, Heatmap illustrating correlation coefficients among the relative proportions of cell subtypes in relation to their corresponding cell types across 86 samples from three studies. We identified clusters/modules by segmenting the hierarchical clustering tree at the red line and assigned annotations based on the modules discovered in Fig. 2D.

Supplementary Figure 7.

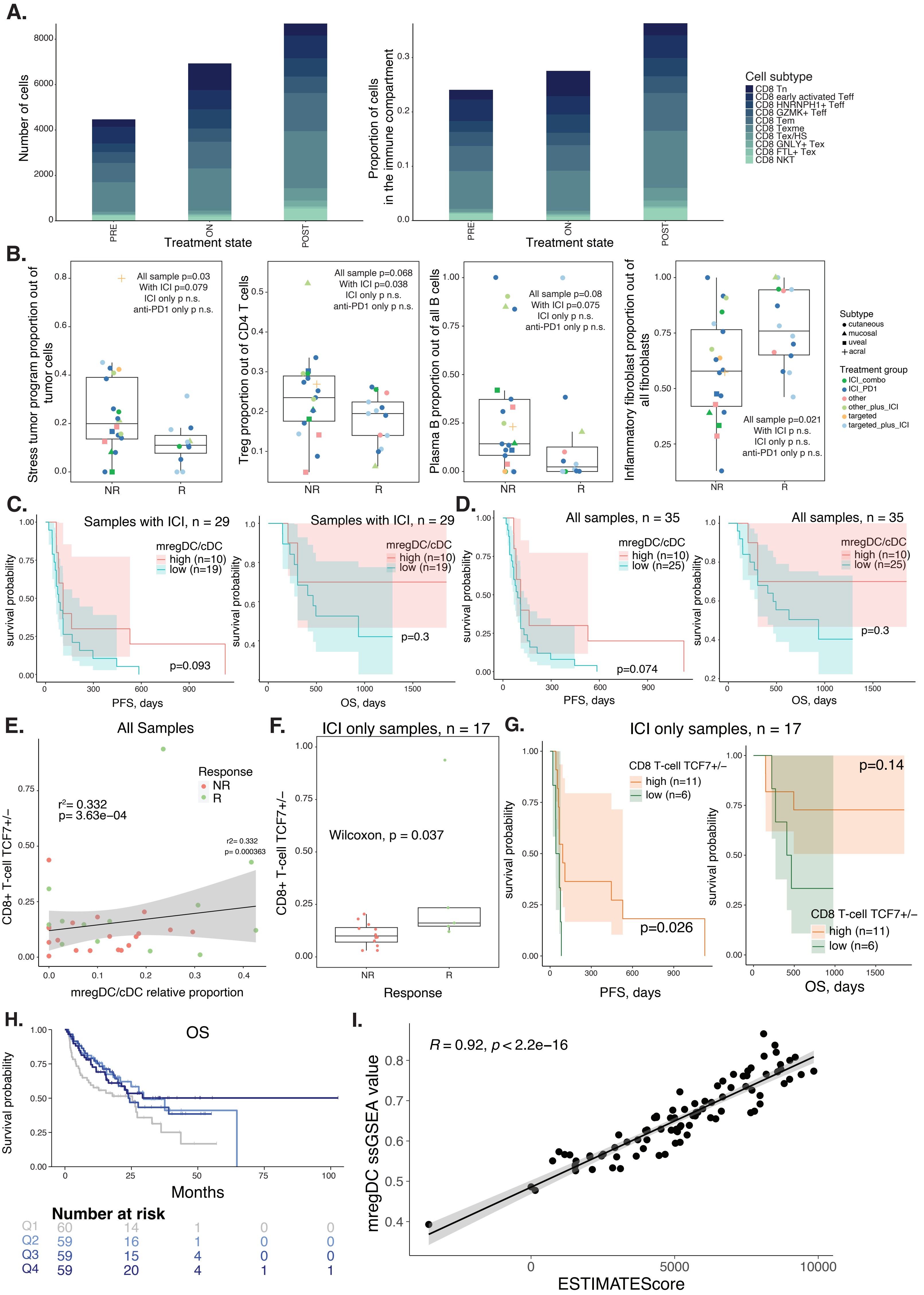

Supplementary Figure 7. Cell subtype association with ICI response and patient survival. A, Barplots showing the average number (left) and proportion of CD8 T-cells in 11 pre-, 13 on-, and 9 post-treatment samples, with the bars filled in by CD8 T-cell subtypes. B, Boxplots comparing relative proportions of selected cell subtypes between 21 non-responders and 14 responders. Each dot represents a sample, with its color corresponding to the treatment group and its shape corresponding to the melanoma subtype. C and D, Survival plots for 29 samples treated with ICI (C) or all 35 samples (D) and split by 20% of mregDC relative proportion. E, Scatterplot correlating mregDC relative proportion and TCF7+ CD8 T ratio using all 35 samples. Each dot corresponds to a sample and is colored by treatment response. F, Boxplot comparing TFC7+ versus TCF7- CD8 T cell ratio between 12 ICI non-responders and 5 ICI responders. G, Survival plots for 17 samples treated with ICI only and split by the median value of TCF7+ versus - CD8 T cell ratio. H, Overall survival plot for the 318 bulk RNA-seq samples split by their mregDC scores. I, Scatterplot correlating ESTIMATE scores and mregDC scores, measured using ssGSEA, in the bulk RNA-seq meta-cohort with 318 samples. P values for boxplots were calculated using the Wilcoxon Rank Sum test. P values for survival plots were calculated using the Log Rank Sum test. R-square and P values for scatterplots were calculated using the Pearson Correlation test. NR, non-responder; R, responder; combo, anti-PD-1+anti-CTLA-4; PFS, progression free survival; OS, overall survival. The boxplots in panels B and F show the distribution of the data, with the central line representing the median (50th percentile), the box indicating the interquartile range (IQR) from the 25th to 75th percentile, and the whiskers extending to the minimum and maximum values within 1.5 times the IQR.

Supplementary Figure 8.

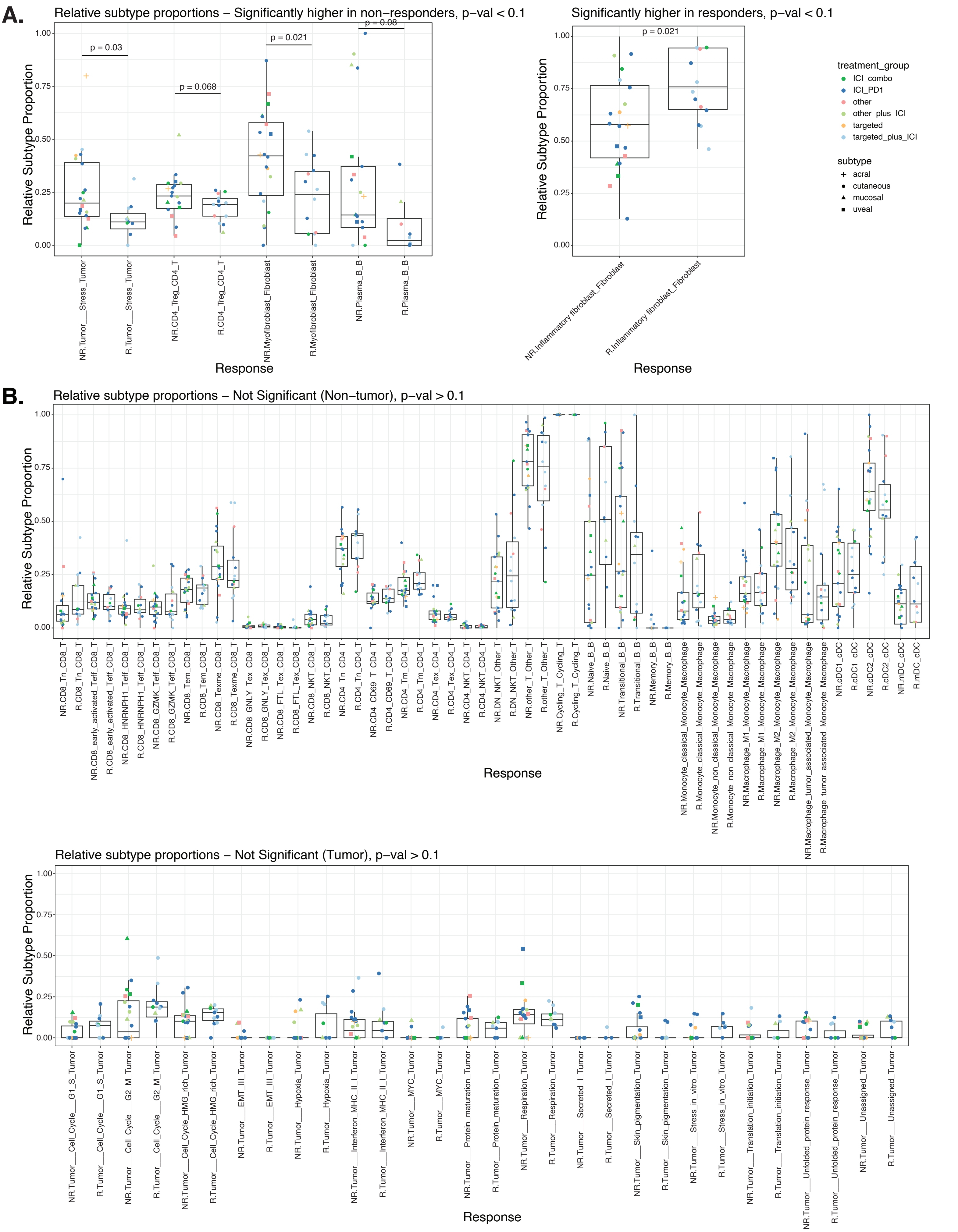

Supplementary Figure 8. Cell subtype association with treatment response. A,B, Boxplots comparing relative proportions of all the cell subtypes between 14 responding and 21 non-responding tumor samples. Comparisons with p values <0.1 are shown in (A) with subtypes showing higher abundance in non-responders on the left and the ones showing higher abundance in responders on the right. Comparisons with p values >0.1 are shown in (B) with non-tumor subtypes on the top and tumor subtypes on the bottom. P values were calculated using the Wilcoxon Rank Sum test. The boxplots in all panels show the distribution of the data, with the central line representing the median (50th percentile), the box indicating the interquartile range (IQR) from the 25th to 75th percentile, and the whiskers extending to the minimum and maximum values within 1.5 times the IQR.

Supplementary Figure 9.

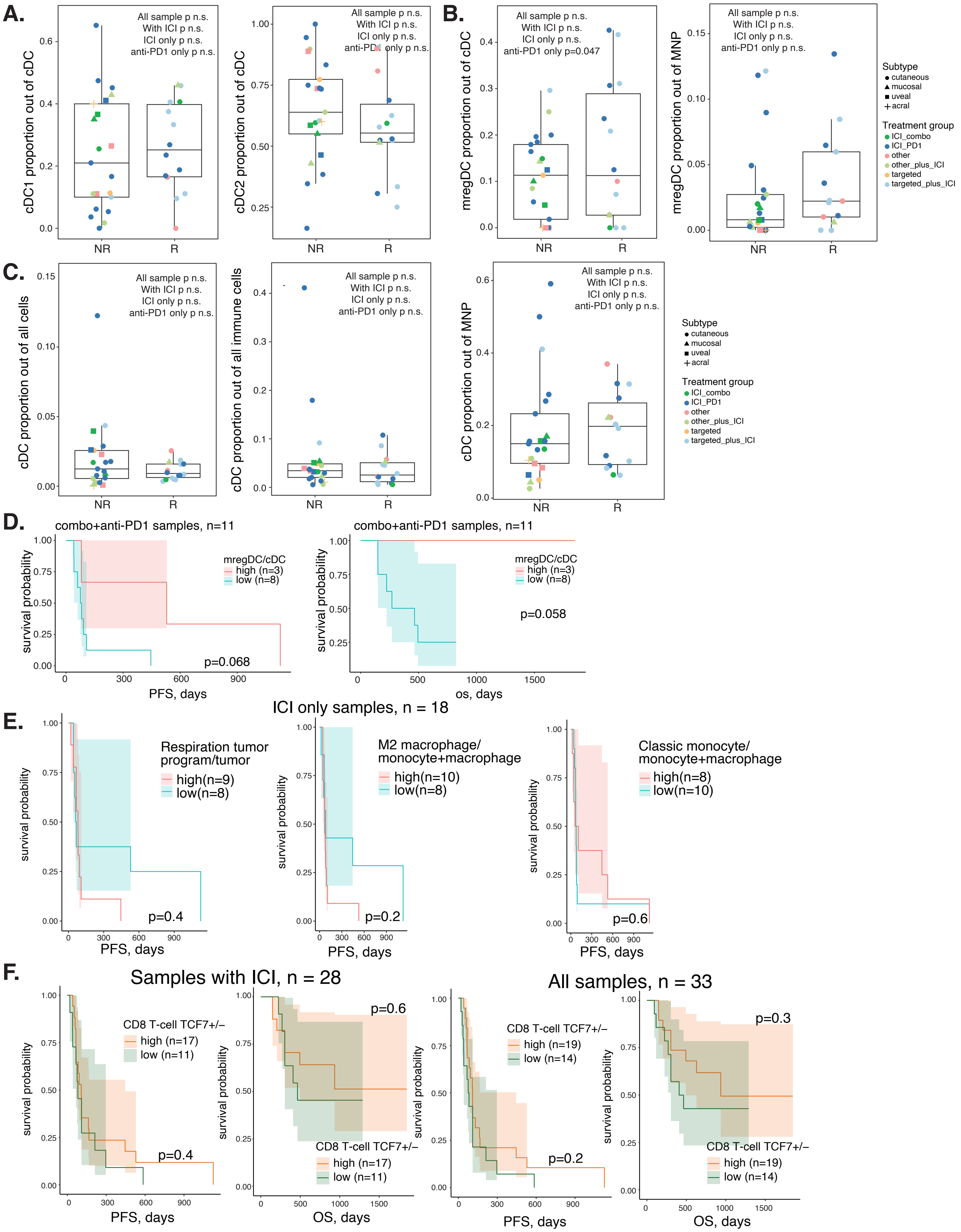

Supplementary Figure 9. Cell subtype relative proportion association with treatment response and patient survival. A-C, Boxplots comparing relative proportions of cDC1 and cDC2 out of all cDC (A), and mregDC out of all cDC or all MNP(B), and proportions of cDC out of all cells, all immune cells or all MNP (C) between 21 non-responders and 14 responders for all 35 samples. Each dot represents a sample, with its color corre-sponding to the treatment group and its shape corresponding to the melanoma subtype. D-F, Survival plots for 11 ICI-only samples split by mregDC including only the latest timepoint sample per patient (D), for 17 ICI-only samples split by selected subtype relative proportions (E), or for 28 samples treated with ICI and all 33 samples split by TCF7+ CD8 T cell ratio (E). P values for boxplots were calculated using the Wilcoxon Rank Sum test. P values for survival plots were calculated using the Log Rank Sum test. NR, non-responder; R, responder; combo, anti-PD-1+anti-CTLA-4; PFS, progression free survival; OS, overall survival; MNP, mononuclear phagocyte. The boxplots in panels A, B, and C show the distribution of the data, with the central line representing the median (50th percentile), the box indicating the interquartile range (IQR) from the 25th to 75th percentile, and the whiskers extending to the minimum and maximum values within 1.5 times the IQR.

Supplementary Figure 10.

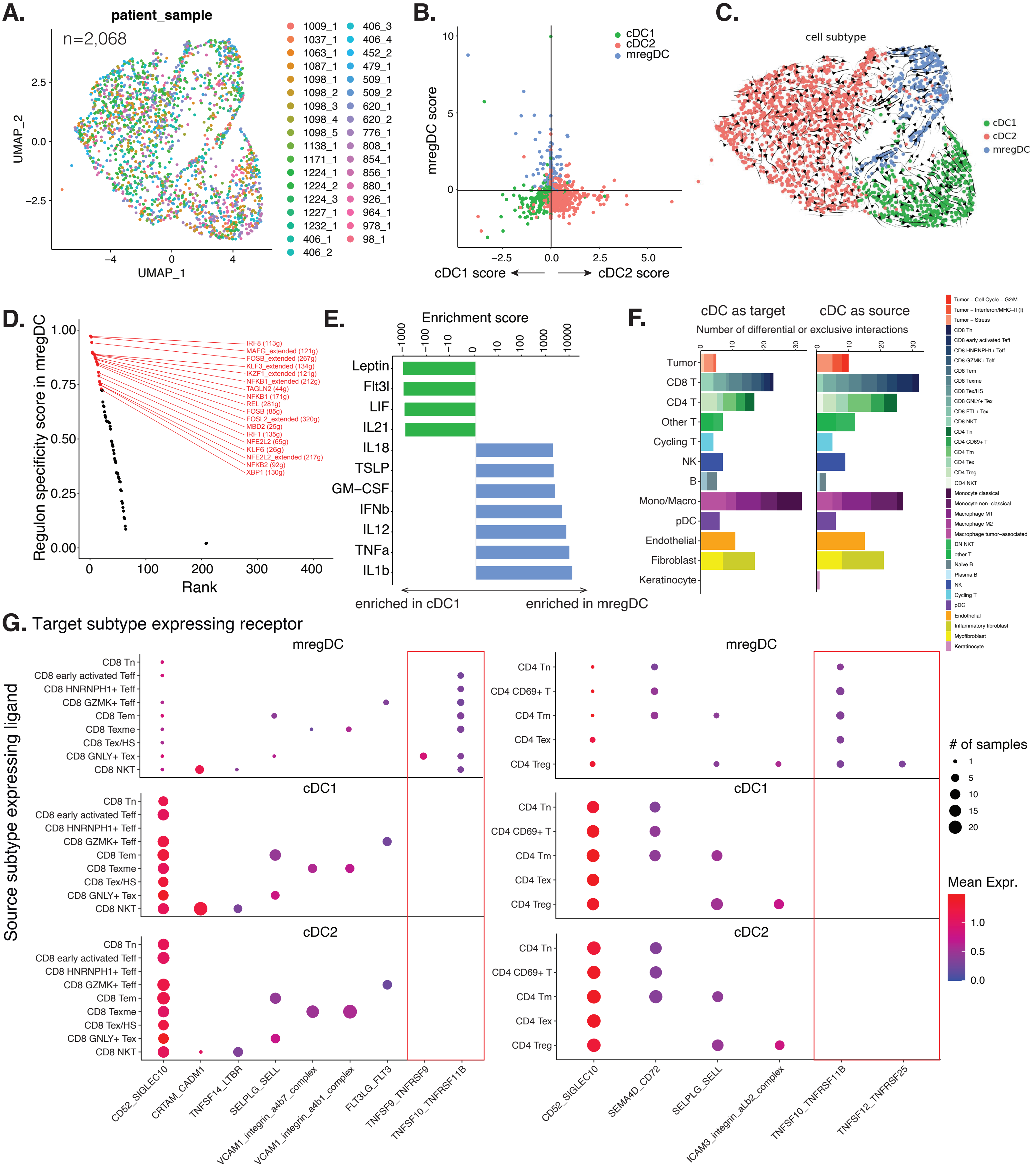

Supplementary Figure 10. Additional transcriptional characterization and cell-cell communication of cDC subtypes. A, UMAP embedding of 2,068 cDCs from 35 samples colored by sample. B, Scatterplot showing cDC1, cDC2 and mregDC scores per cell based on the marker genes reported in Maier et al. C, CytoTrace stream embedding overlaid on UMAP showing converging transition paths from cDC1 and cDC2 to mregDC. Arrows show state transition directions. D, Scatterplot of mregDC regulon specificity scores inferred by SCENIC. Top 20 regulons are high-lighted. E, Barplot showing cytokines with transcriptional signatures enriched in mregDC relative to cDC1, based on Cui et al49. F, Barplots showing the number of differential or exclusive interactions by cell type, with the top panel corresponding to cDC as source and the bottom panel cDC as target. The bars are colored by the subtypes in each cell type. G, Dotplots illustrating inferred cell-cell communications either with differential activi-ties between mregDC and other cDC or exclusively detected in mregDC. The left and right panels plotted inter-actions with cDC expressing receptors and CD8 or CD4 T-cell expressing ligands, respectively.

Supplementary Figure 11.

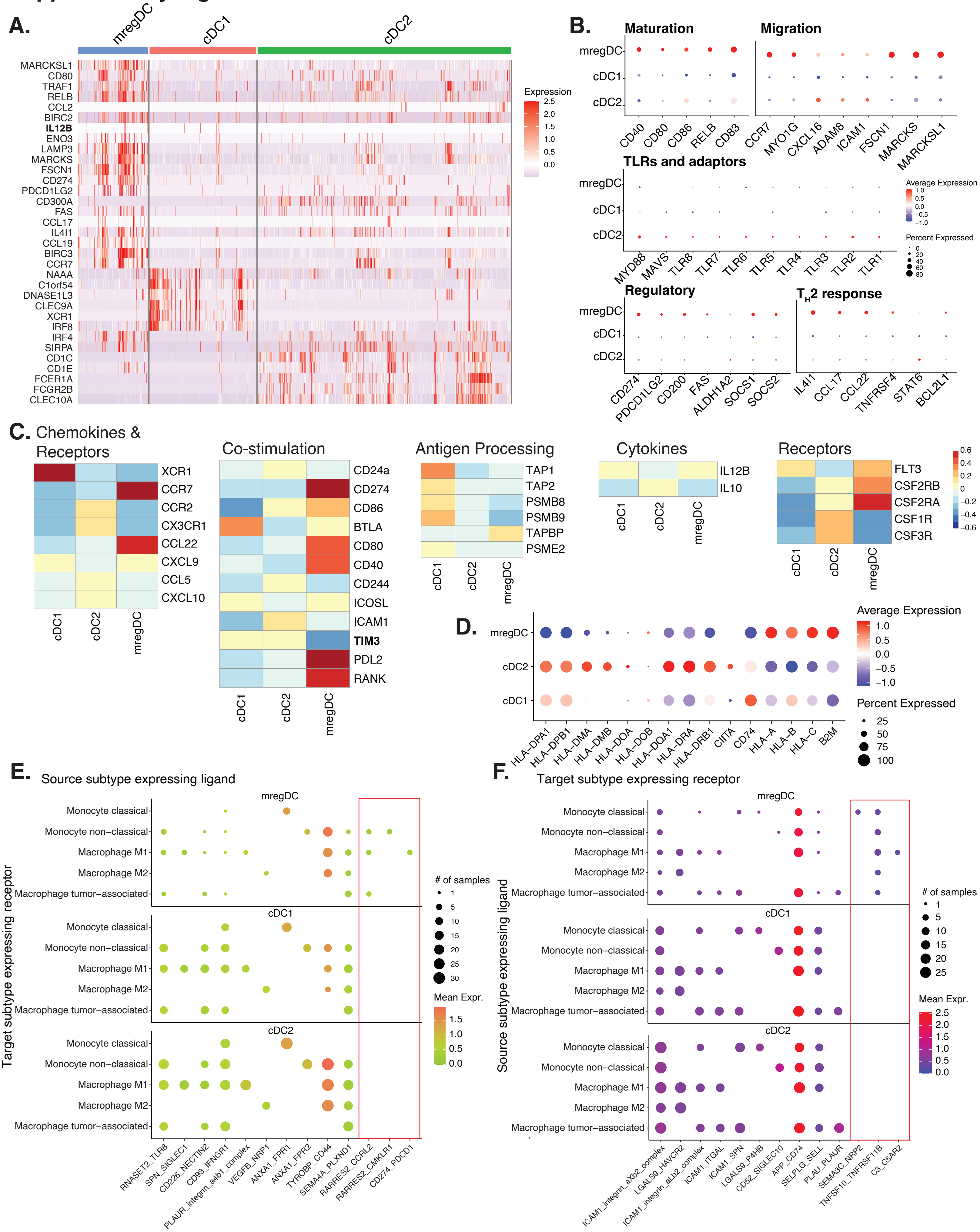

Supplementary Figure 11. Expression patterns of markers reported in other studies and additional cell-cell communication across cDC subtypes. A, Heatmap plotting expression of marker genes reported in Maier et al.[8] in 338 mregDC, 513 cDC1 and 1,217 cDC2. B, Dotplots showing expression patterns of genes grouped by biological processes, as reported in Maier et al.[8], across cDC subtypes. C, Heatmap illustrating average expression of genes for 513 cDC1, 1,217 cDC2 and 338 mregDC. Gene groups were obtained from Broz et al.[9] D, Dotplot showing antigen presentation related gene expression in cDC1, cDC2 and mregDC. E,F, Dotplots illustrating inferred cell-cell communications either with differential activities between mregDC and other cDC or exclusively detected in mregDC, with cDC expressing ligands (E) or receptors (F) and monocyte/macrophage subtypes expressing receptors (E) or ligands (F), respectively. P values for dotplots were calculated using the Wilcoxon Rank Sum test.

Supplementary Figure 12.

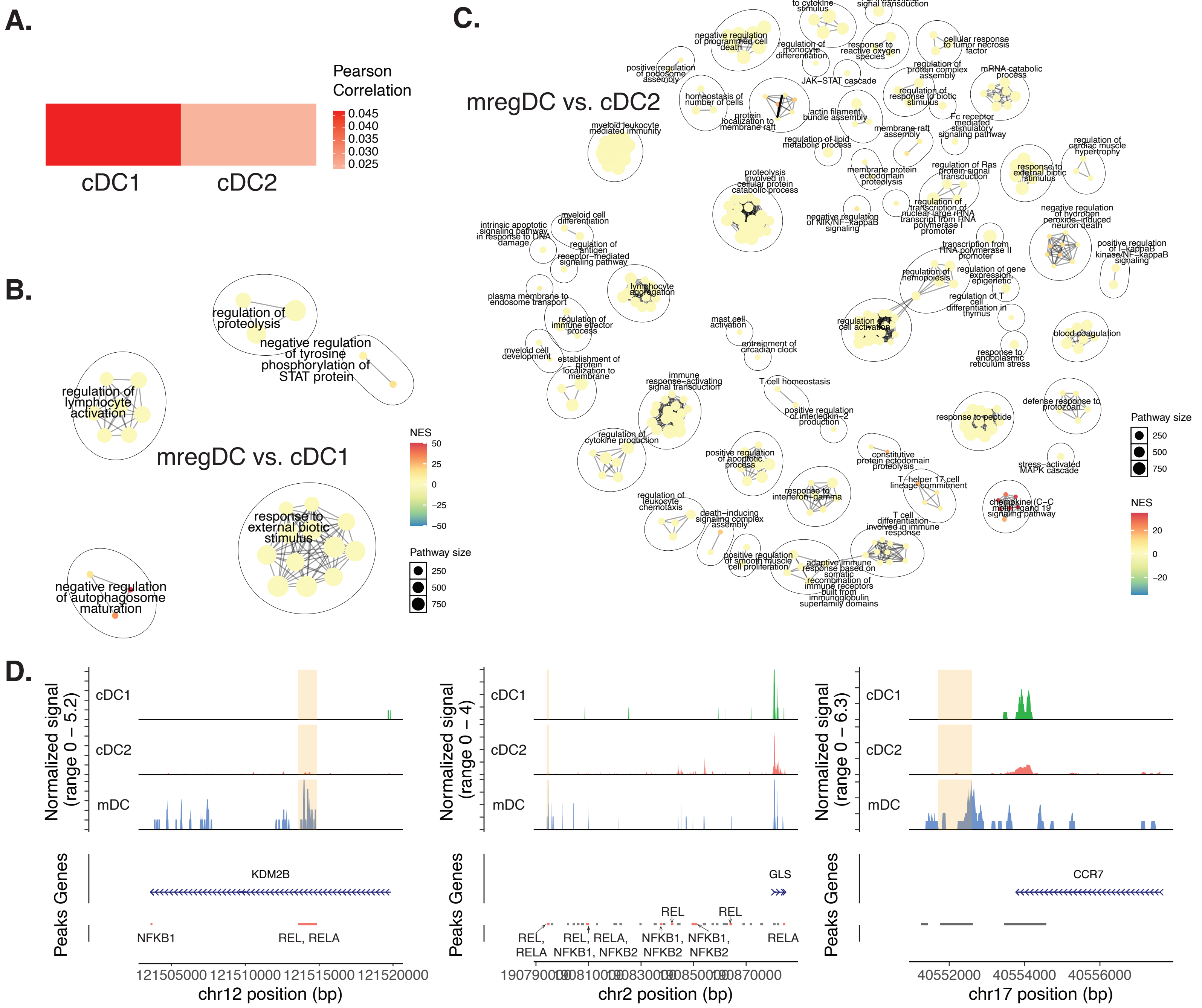

Supplementary Figure 12. Additional epigenomic analysis results for cDC subtypes. A, Overall activity correlation across accessible regions specific to 14 mregDCs in 17 cDC1s and 203 cDC2s. B,C, Gene ontology (GO) networks showing the GO terms enriched by the differentially accessible regions (DARs) between 14 mregDCs and 17 cDC1s (B), and 14 mregDCs and 203 cDC2s (C). The enrichment analysis was carried out using the GREAT program78. D, Track plots comparing normalized number of reads across cDC subtypes (17 cDC1s, 203 cDC2s and 14 mregDCs) underlying enhancers associated with genes important for cDC functions. mDC is short for mregDC in some figure panels due to space constraints. A peak is colored orange if it contains motifs for members of the NF-κB transcription factor family.

Supplementary Figure 13.

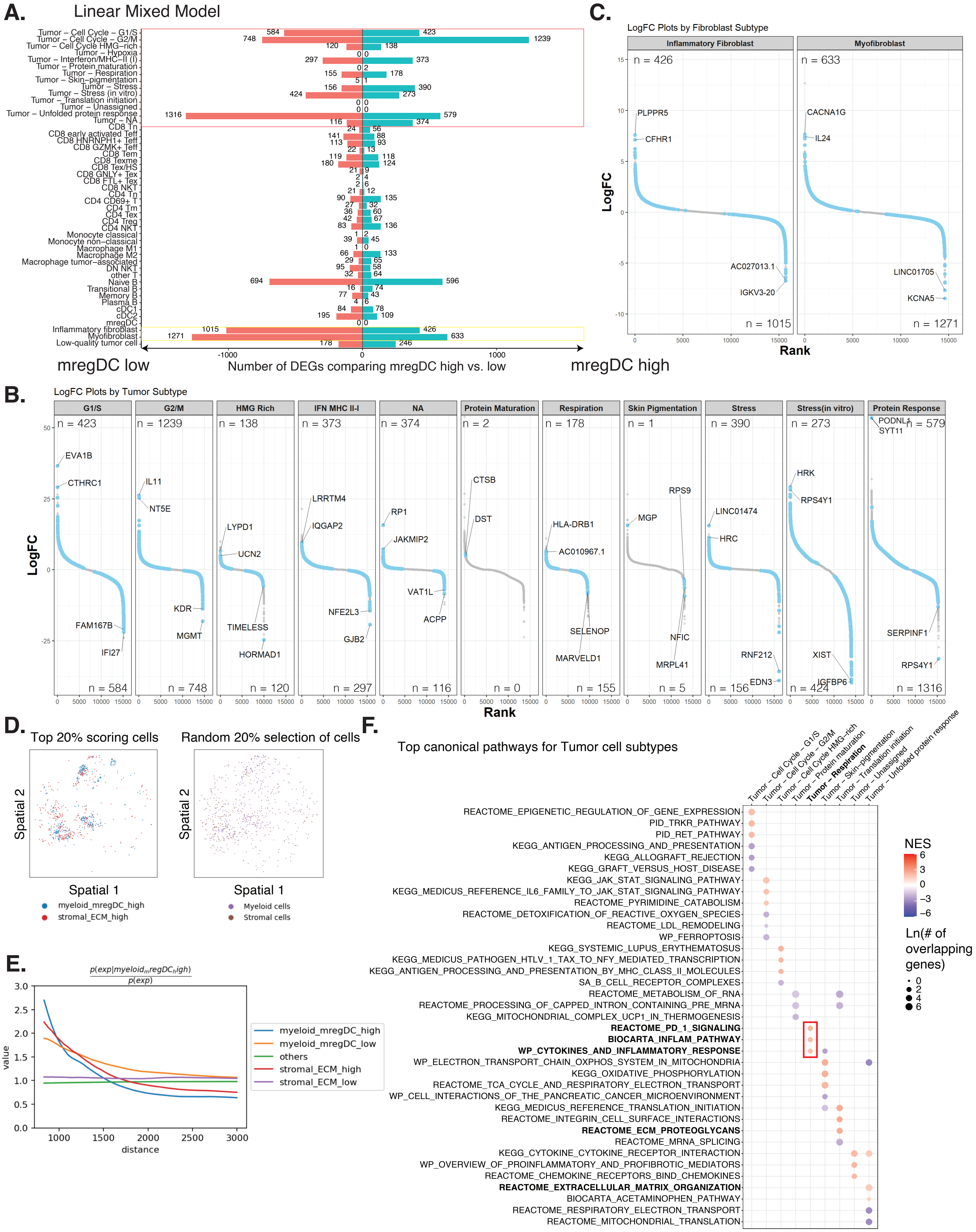

Supplementary Figure 13. Cell subtype-specific factors associated with mregDC proportions. A, Bar plots showing the number of cell subtype-specific differentially expressed genes (DEGs) significantly upregulated or downregulated in 10 mregDC high samples compared to 25 mregDC low samples, detected by a - linear mixed model. B,C, Aggregated rank-logFC plots across tumor programs (B) or fibroblast subtypes (C) showing DEGs passing the adjusted P value threshold in light blue and the rest of genes in gray. The top two genes with large average log2 fold changes in both directions are labeled, respectively. D, Locations of myeloid cells and stromal cells with the top 20% of mregDC scores and ECM scores, respectively (top) or a random 20% selection of cells using a spatial tran-scriptomics slide from Biermann et al.[5] E, Probability of observing 'ECM-high' or 'ECM-low' fibroblasts within a 1000 radius of a 'mregDC-high' cell. Please refer to Materials and Methods for details. F, Dot plots illustrating the top three pathways enriched by the up and downregulated DEGs for each tumor program. The pathway names were in bold if discussed in the text.

Supplementary Figure 14.

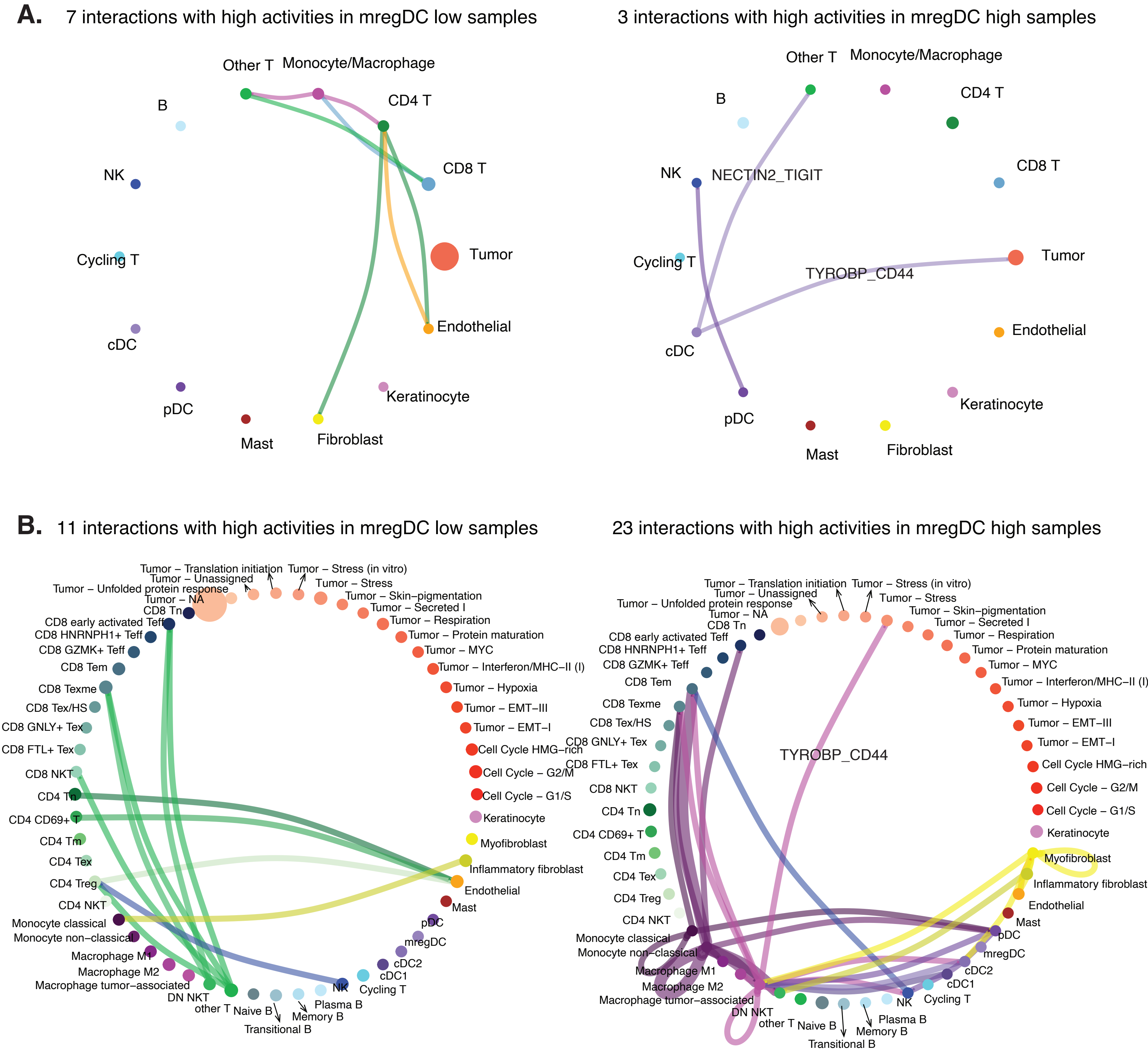

Supplementary Figure 14. Cell-cell communications with differential activities associated with mregDC proportions. A,B, Networks depicting cell-cell interactions, inferred by using CellPhoneDB[10], with differential activities (co-expression patterns) in 10 mregDC high samples vs. 25 mregDC low samples. Nodes represent cell types (A) or subtypes (B). Node sizes correspond to the number of cells in the cell type/subtype within the cohort (mregDC high or low). Edge weights are equal to the number of interactions between each pair of cell types/subtypes.

Supplementary References:

1.Tirosh, I. et al. Dissecting the multicellular ecosystem of metastatic melanoma by single-cell RNA-seq. Science 352, 189–196 (2016).
2.Jerby-Arnon, L. et al. A Cancer Cell Program Promotes T Cell Exclusion and Resistance to Checkpoint Blockade. Cell 175, 984–997.e24 (2018).
3.Sade-Feldman, M. et al. Defining T Cell States Associated with Response to Checkpoint Immunotherapy in Melanoma. Cell 175, 998–1013.e20 (2018).
4.Zhang, C. et al. A single-cell analysis reveals tumor heterogeneity and immune environment of acral melanoma. Nat. Commun. 13, 7250 (2022).
5.Biermann, J. et al. Dissecting the treatment-naive ecosystem of human melanoma brain metastasis. Cell 185, 2591-2608.e30 (2022).
6.Gavish, A. et al. Hallmarks of transcriptional intratumour heterogeneity across a thousand tumours. Nature 618, 598–606 (2023).
7.Rich, A. L., Lin, P., Gamazon, E. R. & Zinkel, S. S. The broad impact of cell death genes on the human disease phenome. Cell Death Dis 15, 251 (2024).
8.Maier, B. et al. A conserved dendritic-cell regulatory program limits antitumour immunity. Nature 580, 257–262 (2020).
9.Broz, M. L. et al. Dissecting the tumor myeloid compartment reveals rare activating antigen-presenting cells critical for T cell immunity. Cancer Cell 26, 638–652 (2014).
10.Efremova, M., Vento-Tormo, M., Teichmann, S. A. & Vento-Tormo, R. CellPhoneDB: inferring cell–cell communication from combined expression of multi-subunit ligand–receptor complexes. Nat. Protoc. 15, 1484–1506 (2020).
