# Supplementary material for: Mature and migratory dendritic cells promote immune infiltration and response to anti-PD-1 checkpoint blockade in metastatic melanoma
Source: Nat Commun. 2025 Sep 1;16:8151. doi: 10.1038/s41467-025-62878-5 (PMC12402436; doi:10.1038/s41467-025-62878-5)
Supplement: Supplementary file 16 — Reporting Summary [file 41467_2025_62878_MOESM16_ESM.pdf]

nature portfolio

Corresponding author(s): Jiekun Yang, Manolis Kellis, Genevieve Boland

Last updated by author(s): Jul 31, 2025

Reporting Summary

Nature Portfolio wishes to improve the reproducibility of the work that we publish. This form provides structure for consistency and transparency in reporting. For further information on Nature Portfolio policies, see our Editorial Policies and the Editorial Policy Checklist.

Statistics

For all statistical analyses, confirm that the following items are present in the figure legend, table legend, main text, or Methods section.

n/a	Confirmed	
☐	☒	The exact sample size (n) for each experimental group/condition, given as a discrete number and unit of measurement
☐	☒	A statement on whether measurements were taken from distinct samples or whether the same sample was measured repeatedly
☐	☒	The statistical test(s) used AND whether they are one- or two-sided<br>*Only common tests should be described solely by name; describe more complex techniques in the Methods section.*
☐	☒	A description of all covariates tested
☐	☒	A description of any assumptions or corrections, such as tests of normality and adjustment for multiple comparisons
☐	☒	A full description of the statistical parameters including central tendency (e.g. means) or other basic estimates (e.g. regression coefficient) AND variation (e.g. standard deviation) or associated estimates of uncertainty (e.g. confidence intervals)
☐	☒	For null hypothesis testing, the test statistic (e.g. F, t, r) with confidence intervals, effect sizes, degrees of freedom and P value noted<br>*Give P values as exact values whenever suitable.*
☒	☐	For Bayesian analysis, information on the choice of priors and Markov chain Monte Carlo settings
☐	☒	For hierarchical and complex designs, identification of the appropriate level for tests and full reporting of outcomes
☐	☒	Estimates of effect sizes (e.g. Cohen's d, Pearson's r), indicating how they were calculated

Our web collection on statistics for biologists contains articles on many of the points above.

Software and code

Policy information about availability of computer code

Data collection	All commercial and open source code are described in the Methods section. Custom code can be found here: https://github.com/KellisLab/scCancer
Data analysis	All commercial and open source code are described in the Methods section. Custom code can be found here: https://github.com/KellisLab/scCancer

For manuscripts utilizing custom algorithms or software that are central to the research but not yet described in published literature, software must be made available to editors and reviewers. We strongly encourage code deposition in a community repository (e.g. GitHub). See the Nature Portfolio guidelines for submitting code & software for further information.

Data

Policy information about availability of data

All manuscripts must include a data availability statement. This statement should provide the following information, where applicable:

- Accession codes, unique identifiers, or web links for publicly available datasets
- A description of any restrictions on data availability
- For clinical datasets or third party data, please ensure that the statement adheres to our policy

The raw and pre-processed scRNA-seq data generated in this study have been deposited in the GEO database under accession code GSE269936 [https://www.ncbi.nlm.nih.gov/geo/query/acc.cgi?acc=GSE269936]. The raw and pre-processed snATAC-seq data for cDCs generated in this study have been deposited in the GEO database under accession code GSE303948 [https://www.ncbi.nlm.nih.gov/geo/query/acc.cgi?acc= GSE303948]. The processed scRNA-seq and snATAC-seq

data are deposited in the Zenodo database under accession code 15603513 [https://doi.org/10.5281/zenodo.15603513]. The scRNA-seq publicly available data used in this study are available in the GEO database under accession code GSE72056 [https://www.ncbi.nlm.nih.gov/geo/query/acc.cgi?acc=GSE72056]7, GSE115978 [https://www.ncbi.nlm.nih.gov/geo/query/acc.cgi?acc=GSE115978]8, GSE120575 [http://www.ncbi.nlm.nih.gov/geo/query/acc.cgi?acc=GSE120575]9, GSE215119 [https://www.ncbi.nlm.nih.gov/geo/query/acc.cgi?acc=GSE215119]10, and GSE200278 [https://www.ncbi.nlm.nih.gov/geo/query/acc.cgi?acc=GSE200278]11. The meta-RNA-seq publicly available data used in this study are available in supplementary tables of the corresponding study or as .rds objects available on GitHub [https://github.com/davidliu-lab/diffIO-hypoxia-figures]42. The remaining data are available within the Article, Supplementary Information, Source Data file or Supplementary Data files.

Research involving human participants, their data, or biological material

Policy information about studies with human participants or human data. See also policy information about sex, gender (identity/presentation), and sexual orientation and race, ethnicity and racism.

Reporting on sex and gender	Samples from 14 females and 22 males were collected. Our study focuses on melanoma, and while it is important to consider sex-specific differences, the sample size for our research is limited. Given the small number of participants, performing a sex-specific analysis would reduce the statistical power and reliability of our findings.
Reporting on race, ethnicity, or other socially relevant groupings	We did not collect and report on race, ethnicity, or other socially relevant groupings for this study.
Population characteristics	We included age, treatment categories, treatment stage, progression-free survival and overall survival data in this study. These population characteristics have been properly corrected in our statistical models.
Recruitment	The metastatic melanoma samples for this study were obtained from Mass General Brigham by G.M.B. under the protocol 11-181. This protocol was approved by the Dana-Farber Cancer Institute Institutional Review Board (PI: G.M.B.). No self-selection bias or other biases were identified.
Ethics oversight	Mass General Brigham

Note that full information on the approval of the study protocol must also be provided in the manuscript.

Field-specific reporting

Please select the one below that is the best fit for your research. If you are not sure, read the appropriate sections before making your selection.

[x] Life sciences [ ] Behavioural & social sciences [ ] Ecological, evolutionary & environmental sciences

For a reference copy of the document with all sections, see nature.com/documents/nr-reporting-summary-flat.pdf

Life sciences study design

All studies must disclose on these points even when the disclosure is negative.

Sample size	The sample size was chosen based on previous single-cell studies.
Data exclusions	Data from patients involved in unpublished clinical trials at the time of sample collection were excluded from the analyses.
Replication	We validated our finding in an independent meta-cohort. All attempts at validation were successful.
Randomization	Covariates were controlled by linear-mixed models.
Blinding	The investigators were not blinded to group allocation during data collection and analysis. In our study, blinding was not implemented because it did not affect the analysis and interpretation of the results.

Reporting for specific materials, systems and methods

We require information from authors about some types of materials, experimental systems and methods used in many studies. Here, indicate whether each material, system or method listed is relevant to your study. If you are not sure if a list item applies to your research, read the appropriate section before selecting a response.

Materials & experimental systems

n/a	Involved in the study
	[x] Antibodies
[x]	Eukaryotic cell lines
[x]	Palaeontology and archaeology
[x]	Animals and other organisms
[x]	Clinical data
[x]	Dual use research of concern
[x]	Plants

Methods

n/a	Involved in the study
[x]	ChIP-seq
[x]	Flow cytometry
[x]	MRI-based neuroimaging

Antibodies

Antibodies used

rabbit anti-human CCR7 (1:200, Proteintech), Armenian hamster anti-human CD11c (1:100, Novus Bio, clone AP-MAB0806), mouse anti-human IRF4 (1:300, Thermo, clone 3B1D2), polyclonal goat anti-human IRF8 (1:200, Novus Bio), goat anti-rabbit AF750 (1:2000, Thermo), goat anti-armenian hamster AF488 (1:1000, Thermo), goat anti-mouse IgG2a AF594 (1:2000, Thermo) and donkey anti-goat AF555 (1ug/ml, Thermo)

Validation

IRF8 antibody, Cat#- NB100-1093, polyclonal, Novus Biologicals
Species Reactivity – Human
Application - western blot, IHC/IF, ELISA
Validation – The IRF8 primary antibody has been validated by the manufacturer using paraffin embedded human tonsil tissue at 5ug/ml concentration.

MUM1(IRF4) antibody, Cat#- 60353-1-IG, clone-3B1D2, Proteintech via Thermo
Species Reactivity – Human
Application - WB, IHC, ICC/IF
Validation – The IRF4 primary antibody has been validated by the manufacturer using PFA fixed HeLa cells and has also been validated in FFPE human tissues for IHC in publication (..) (Qi Z, Duan L, Yuan G, Liu J, Li J, Li G, Yu Y, Xu Y, Ma S, Pan Y, Zhang Y. Clinical Impact of the Histopathological Index and Neuroimaging Features Status in Primary Central Nervous System Diffuse Large B-Cell Lymphoma: A Single-Center Retrospective Analysis of 51 Cases. Front Oncol. 2022 Jul 8;12:769895. doi: 10.3389/fonc.2022.769895. PMID: 35875161; PMCID: PMC9304881.)

CCR7 antibody, Cat#- 25898-1-AP, polyclonal, Proteintech
Species Reactivity – Human, mouse and rat
Application - WB, IHC, IIF, ELISA
Validation – This antibody has been validated for immunofluorescence on FFPE human skin tissue and has been cited in a published study. (…) Shan M, Liu H, Hao Y, Meng T, Feng C, Song K, Wang Y. IL-4 and CCR7 play an important role in the development of keloids in patients with a family history. Am J Transl Res. 2022 May 15;14(5):3381-3394. PMID: 35702126; PMCID: PMC9185030.

CD11c antibody, Cat#- NB110-97871, clone-AP-MAB0806, Novus Biologicals
Species Reactivity – Human and mouse
Application – Flow, WB, IHC, ICC/IF, IP, CyTOF
Validation – This antibody has been validated in multiple publications particularly against human species for immunofluorescence in (…). Jang YJ, Lim JY, Kim S, Lee Y, Kweon MN, Kim JH. Enhanced Interferon-β Response Contributes to Eosinophilic Chronic Rhinosinusitis. Front Immunol. 2018 Oct 16;9:2330. doi: 10.3389/fimmu.2018.02330. PMID: 30455684; PMCID: PMC6232691..

Plants

Seed stocks

Report on the source of all seed stocks or other plant material used. If applicable, state the seed stock centre and catalogue number. If plant specimens were collected from the field, describe the collection location, date and sampling procedures.

Novel plant genotypes

Describe the methods by which all novel plant genotypes were produced. This includes those generated by transgenic approaches, gene editing, chemical/radiation-based mutagenesis and hybridization. For transgenic lines, describe the transformation method, the number of independent lines analyzed and the generation upon which experiments were performed. For gene-edited lines, describe the editor used, the endogenous sequence targeted for editing, the targeting guide RNA sequence (if applicable) and how the editor was applied.

Authentication

Describe any authentication procedures for each seed stock used or novel genotype generated. Describe any experiments used to assess the effect of a mutation and, where applicable, how potential secondary effects (e.g. second site T-DNA insertions, mosiacism, off-target gene editing) were examined.
